# A Rare Case of Viral Meningitis and Probable Metabolic Encephalopathy in a Renal Transplant Patient

**DOI:** 10.7759/cureus.76602

**Published:** 2024-12-30

**Authors:** Vijay Sinha, Krishna Patel, Krithika Giresh, Alexia McDonough, Gagandeep S Grewal

**Affiliations:** 1 Internal Medicine, Merit Health Wesley, Hattiesburg, USA; 2 Internal Medicine, William Carey University College of Osteopathic Medicine, Hattiesburg, USA

**Keywords:** aseptic encephalitis, immunocompromised patient, metabolic encephalopathy, renal transplant, renal transplant patient, west nile encephalitis

## Abstract

This case report highlights the complex clinical presentation of a 43-year-old male with a history of renal transplantation, hypertension, and diabetes mellitus, who developed viral meningitis with probable metabolic encephalopathy. The multidisciplinary approach involved infectious disease specialists, transplant nephrologists, and neurologists. This case provides unique learning points such as highlighting the complexities of diagnosing and managing viral meningitis in an immunocompromised post-transplant patient, emphasizing the importance of a multidisciplinary approach, innovative medication delivery, and awareness for complications such as metabolic encephalopathy and persistent fevers. The patient’s management was complicated by altered mental status, high fevers, and medication delivery challenges, necessitating a transfer to a higher-level care facility.

## Introduction

Aseptic meningitis is an inflammatory condition marked by inflammation of the meninges, characterized by a negative cerebrospinal fluid (CSF) bacterial culture and elevated white blood cell (WBC) counts in the CSF [1]. Common symptoms include severe headache, stiff neck, photophobia, nausea, vomiting, and altered mental status [1].

Direct embolization to meningeal arteries causes meningoencephalitis, which is followed by an invasion of the infecting organism's parenchyma or CSF [2]. Hematogenous seeding is the first of two inoculation routes that causes meningitis. Following mucosal invasion, bacteria colonize the nasopharynx, enter the bloodstream, travel to the subarachnoid space, and then cross the blood-brain barrier to directly cause an inflammatory and immune-mediated response [2]. The second method of inoculation is direct contiguous spread, in which pathogens can enter the CSF by surgical operations, penetrating trauma, foreign items such as medical devices, or adjacent anatomic structures such as sinusitis and otitis media [2]. Direct viral invasion or a hypersensitive reaction to a virus or another foreign protein can result in encephalitis, which is a post-infectious immunologic consequence [2]. These viruses can be sporadic, such as varicella-zoster virus and herpes simplex, or epidemic, such as poliovirus [2].

## Case presentation

A 43-year-old Caucasian male, with a weight of 90.3 kilograms, a height of 190.5 centimeters, and a history of renal transplant six years ago, hypertension, and type II diabetes mellitus presented to the hospital with a two-day history of headache, neck pain, and photophobia. Renal antirejection medications include tacrolimus 0.5 mg oral three capsules daily and CellCept 360 mg oral delayed released two tablets twice daily. The patient’s type II diabetes mellitus medications include dapagliflozin 10 mg oral tablet daily, glimepiride 4 mg oral tablet daily, and sitagliptin 100 mg oral tablet daily. The patient takes medications for his high blood pressure but states that his wife administers the medication to him daily. He reported a history of migraines but was not on prophylactic treatment. The patient denied nausea, vomiting, changes in vision, trauma, abdominal pain, or urinary symptoms. On initial physical examination, he appeared drowsy and somnolent, with a headache radiating down his neck and a fever of 100.1°F. Initial vital signs included a blood pressure of 178/84 mmHg, a heart rate of 115 beats per minute (bpm), and an oxygen saturation of 94% on room air. The patient’s total admission course was six days.

**Figure 1:**
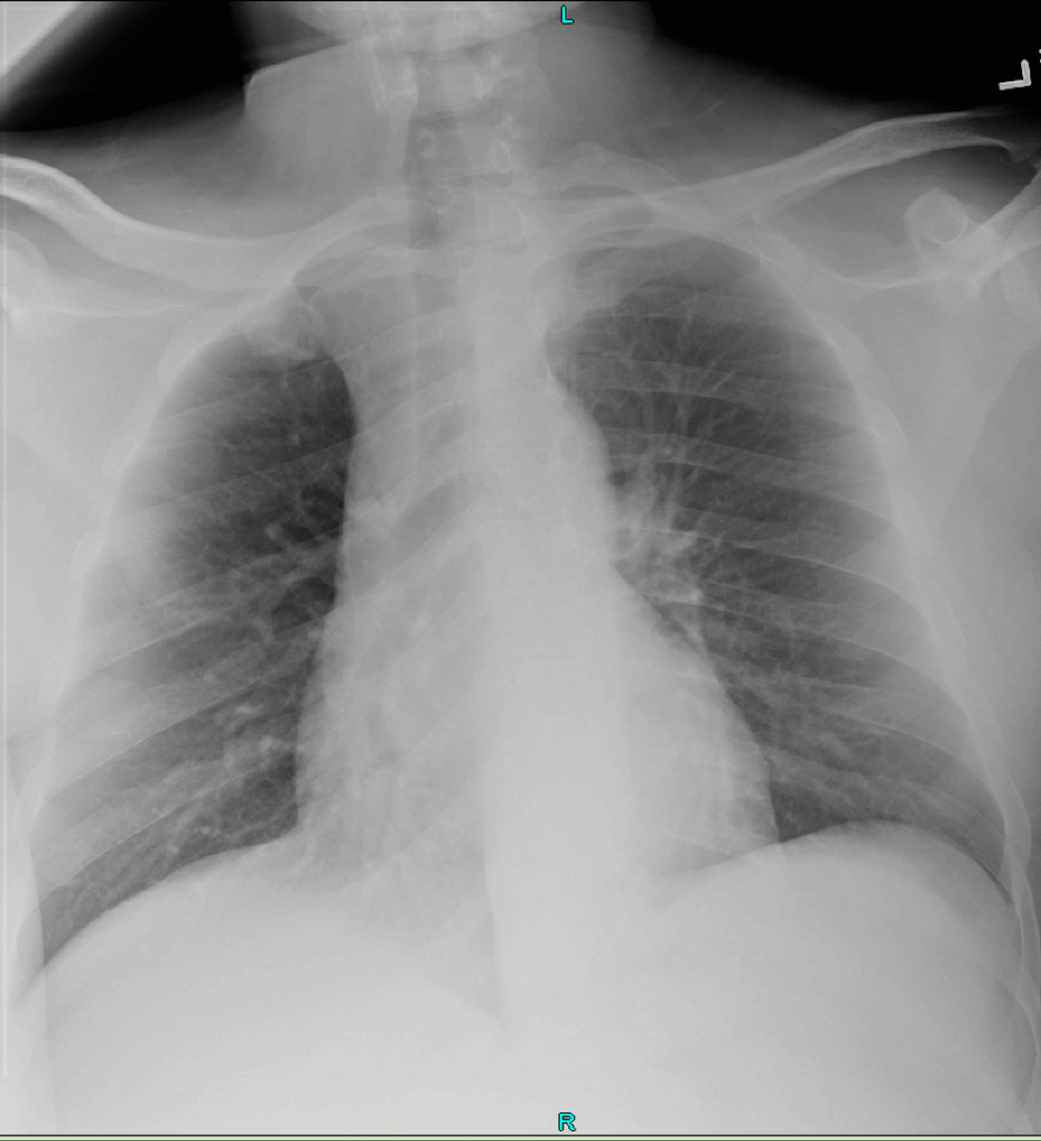
Chest X-ray (1 view) Heart size is normal. Vasculature is normal. The lungs are clear. The mediastinal structures and bony thorax show no acute abnormality.

**Figure 2:**
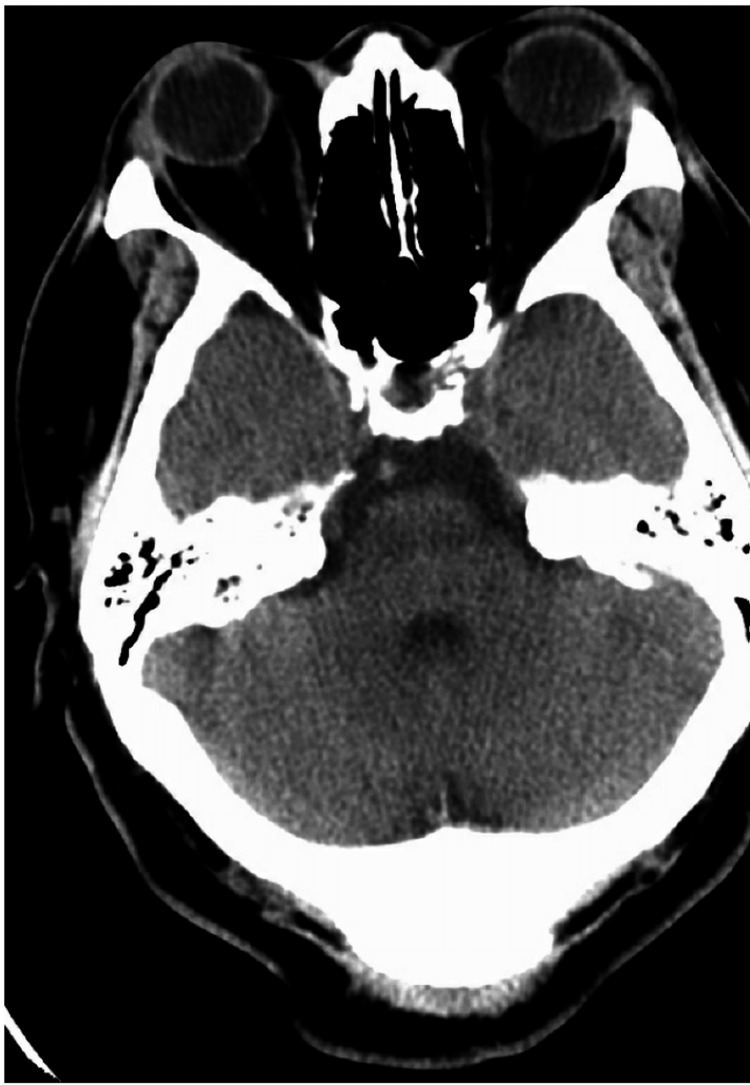
Non-contrast computed tomography of the head There is no acute hemorrhage, hydrocephalus, or herniation. Gray-white differentiation is preserved. Ventricles, sulci, and cisterns are normal.

Initial investigations showed no signs of infectious pathology on chest X-ray (Figure 1). A lumbar puncture revealed a CSF protein level of 107.1 mg/dL, a glucose level of 69 mg/dL, a white blood count (WBC) of 57/mm³, and Gram stain findings of rare WBCs. Blood and CSF cultures were negative to date, and a viral panel for influenza A/B and COVID-19 was negative (Table 1). The elevated CSF protein and WBC levels, together with a normal CSF glucose level and negative cultures, clearly suggested viral meningitis, emphasizing the necessity for additional diagnostic tests and multidisciplinary therapy to address potential secondary problems in an immunocompromised patient. An initial computed tomography (CT) scan of the head without contrast showed no acute intracranial abnormalities (Figure 2).

**Table 1:** Lumbar puncture, viral panel, and blood culture results on admission CSF, cerebrospinal fluid; HSV, herpes simplex virus; RT-PCR, reverse transcription polymerase chain reaction; WBC, white blood cell

Test/Parameter	Result	Normal Range
CSF protein	107.1 mg/dL	15-45 mg/dL
CSF glucose	69 mg/dL	40-70 mg/dL
CSF WBC	57 cells/µL	0-5 cells/µL
CSF culture with gram stain	Rare WBCs	No organisms seen
HSV 1 and 2 DNA Quant (CSF RT-PCR)	Pending	Negative
West Nile & arbovirus panel (CSF)	Pending	Negative
Influenza A and B	Negative	Negative
COVID-19 SARS-CoV-2 antigen	Negative	Negative
Blood cultures	Negative	Negative

The patient was treated empirically with intravenous (IV) acyclovir (900 mg every 8 hours) for suspected viral meningitis. While there was initial improvement, he developed recurrent high fevers requiring ice packs, nonsteroidal anti-inflammatory drugs (NSAIDs), and antipyretics. Broad-spectrum antibiotics, including vancomycin, ceftriaxone, and antifungal treatment with micafungin were added due to continued fever and clinical decline. By DAY 3, his mental status worsened despite improving renal function with IV fluids. Metabolic encephalopathy was suspected, though ammonia levels were normal at 19 µmol/L.

**Figure 3:**
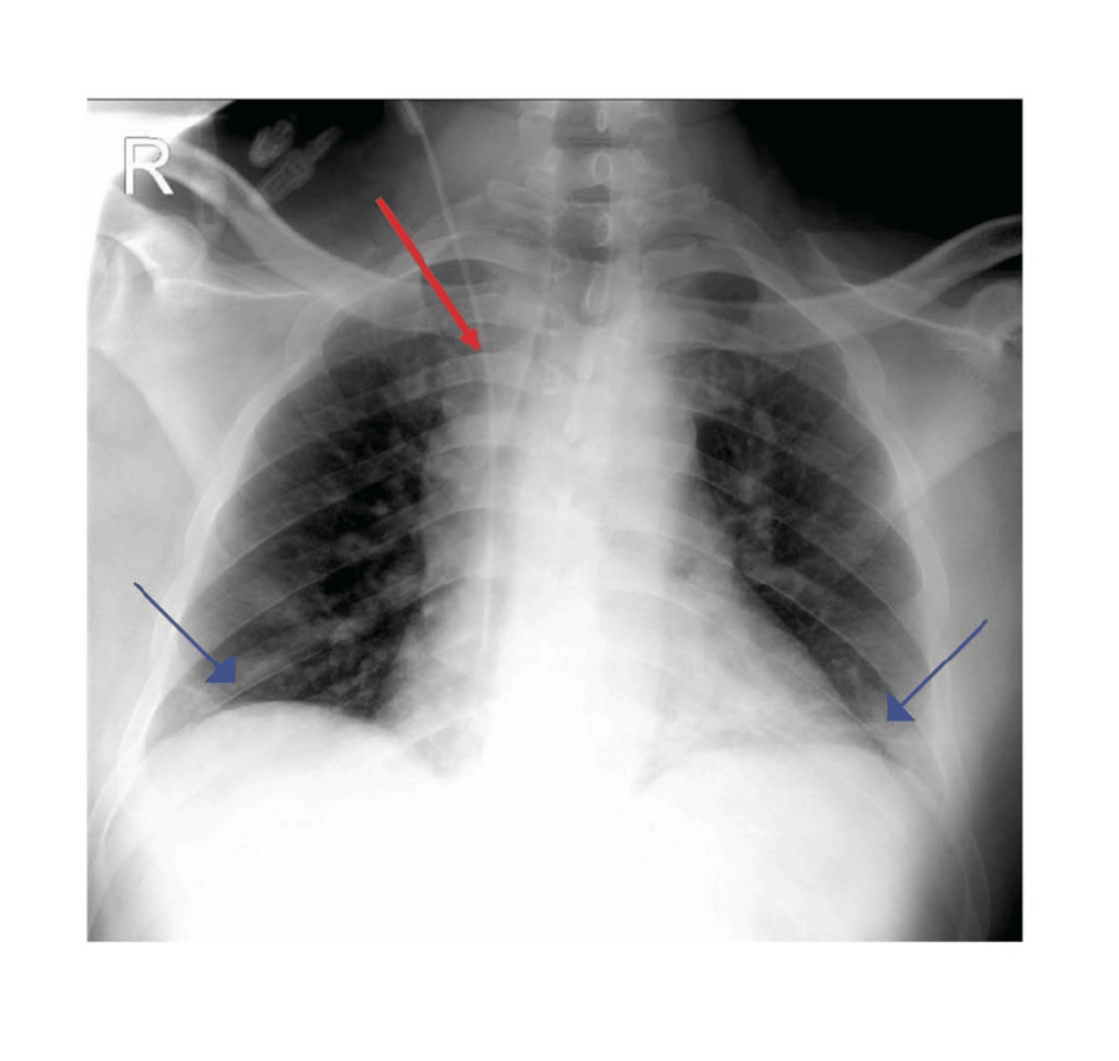
Chest X-ray (1 view) The red arrow indicates the right internal jugular central line with the tip overlying the right atrium. The heart size is unchanged. The blue arrows indicate mild bibasilar opacities. No pneumothorax or large pleural effusion are present. There is no acute osseous abnormality.

Due to the patient’s altered mental status and inability to take medications orally, an oral gastric tube was placed for the administration of tacrolimus and CellCept. Nephrology and transplant surgery consultations ensured the continuation of immunosuppressive therapy, while nutrition services recommended tube feeding for poor oral intake. Persistent fevers and altered mental status necessitated restraints to prevent the removal of medical devices (Table 2). A right internal jugular (IJ) central venous catheter was placed for reliable IV access and medication delivery. A chest X-ray confirmed the right IJ central line placement with the tip at the right atrium and showed mild bibasilar opacities without pneumothorax or effusion (Figure 3). The patient was placed on the transfer list to a higher-level care facility and was accepted overnight. Given that the patient was transferred to a different facility, we were unable to follow up with the results of the MRI. ten days after transfer to an outside facility, on September 12, 2024, the patient tested positive for West Nile virus.

**Table 2:** Persistent fevers throughout admission

Time	Value
9/2/2024 04:00 CDT	102.7°F
9/2/2024 02:01 CDT	
9/2/2024 01:51 CDT	
9/2/2024 00:00 CDT	100.3°F
9/1/2024 20:41 CDT	
9/1/2024 20:39 CDT	101.2°F
9/1/2024 20:31 CDT	
9/1/2024 16:00 CDT	100.6°F
9/1/2024 12:00 CDT	101.2°F
9/1/2024 08:00 CDT	103.3°F
9/1/2024 04:00 CDT	102.2°F
9/1/2024 01:00 CDT	100.1°F
9/1/2024 00:00 CDT	103.1°F
8/31/2024 20:00 CDT	100.7°F
8/31/2024 17:15 CDT	101.1°F
8/31/2024 16:00 CDT	99.9°F
8/31/2024 13:00 CDT	106°F
8/31/2024 12:36 CDT	101.5°F
8/31/2024 09:39 CDT	101.2°F
8/31/2024 07:00 CDT	100.7°F
8/31/2024 05:00 CDT	100.4°F
8/31/2024 04:00 CDT	102.6°F
8/31/2024 00:00 CDT	102.9°F
8/30/2024 23:00 CDT	
8/30/2024 20:00 CDT	100.4°F
8/30/2024 19:47 CDT	102.5°F
8/30/2024 15:00 CDT	103.0°F
8/30/2024 13:42 CDT	100.5°F
8/30/2024 12:00 CDT	
8/30/2024 08:00 CDT	101.5°F
8/30/2024 04:00 CDT	98.4°F
8/30/2024 00:00 CDT	102.6 °F
8/29/2024 20:00 CDT	98.0°F
8/29/2024 18:48 CDT	101.0°F
8/29/2024 18:34 CDT	
8/29/2024 15:30 CDT	102.1°F
8/29/2024 12:25 CDT	102.0°F

## Discussion

Humans contract the West Nile virus when bitten by an infected female mosquito [3]. When a mosquito bites an infected bird, it contracts the virus [3]. The two birds most frequently associated with the virus are crows and jays [3]. However, the virus can potentially infect at least 110 additional bird species [3]. The West Nile virus does not transfer from person to person [3]. However, in a few instances, organ donations have caused it to spread [4]. The likelihood of contracting the virus from an organ is uncertain [4]. Not every organ donor undergoes a West Nile virus test [4]. Every blood sample is examined for viruses [4]. Compared to not having any surgery that would need a blood transfusion, the risk of contracting the West Nile virus from blood is significantly lower [4].

Risk factors for meningitis include chronic medical disorders such as renal failure, diabetes, adrenal insufficiency, cystic fibrosis; extremes of age; and a lack of appropriate vaccinations [5]. In addition, immunosuppressed states (iatrogenic, transplant recipients, congenital immunodeficiencies, AIDS) and living in crowded conditions are also important risk factors to consider [5].

Management typically involves supportive care, including pain control, adequate hydration, and close monitoring [6]. Treatment can be particularly challenging in immunocompromised individuals, such as those with renal transplants, malignancies, or autoimmune disorders [7]. We initiate suppressive therapy with acyclovir (400 mg orally twice daily) or valacyclovir (500 mg orally once daily) [8]. The dose of valacyclovir can be increased to 1 g daily for those with breakthrough recurrences [9]. Dose adjustments for patients with reduced kidney function are described in the drug information topics within UpToDate [9]. Long-term use of these agents appears safe, and specific laboratory monitoring of treatment is not recommended [9]. The need to continue suppressive therapy should be evaluated annually; however, patients should be counseled that episodes may recur once the antivirals are stopped [9]. This case highlights the complexities of managing transplant patients with viral infections and encephalopathy, emphasizing the importance of balancing immunosuppression and infection control, addressing unique medication delivery challenges, and taking a multidisciplinary approach.

## Conclusions

This case underscores the complexity of managing viral meningitis and metabolic encephalopathy in a post-renal transplant patient. The altered mental status posed challenges for immunosuppressive therapy and nutritional management. Early involvement of multidisciplinary teams and timely transfer to a higher-level care facility were crucial for addressing complications, including persistent fever and neurological decline. The case also highlights the importance of maintaining a high index of suspicion for secondary infections in immunocompromised patients. Balancing antirejection therapy with infection management is crucial for positive results. Multidisciplinary coordination is essential to optimize outcomes, by ensuring effective infection control, organ preservation, and patient stability and safety.
